# N-myc downstream regulated gene 1 suppresses osteoblast differentiation through inactivating Wnt/β-catenin signaling

**DOI:** 10.1186/s13287-022-02714-5

**Published:** 2022-02-04

**Authors:** Xiaoli Shi, Yunzhu Cen, Liying Shan, Lijie Tian, Endong Zhu, Hairui Yuan, Xiaoxia Li, Ying Liu, Baoli Wang

**Affiliations:** 1 NHC Key Lab of Hormones and Development and Tianjin Key Lab of Metabolic Diseases, Chu Hsien-I Memorial Hospital and Institute of Endocrinology, Tianjin Medical University, Tianjin, 300134 China; 2 College of Basic Medical Sciences, Tianjin Medical University, Tianjin, 300070 China; 3 Stomatological Hospital, Tianjin Medical University, Tianjin, 300070 China

**Keywords:** Adipocyte, Differentiation, N-myc downstream regulated gene 1, Osteoblast, Wnt/β-catenin

## Abstract

**Background:**

N-myc downstream regulated gene 1 (NDRG1) plays a role in a variety of biological processes including differentiation of osteoclasts. However, it is not known if and how NDRG1 regulates osteogenic differentiation of marrow stromal progenitor cells.

**Methods:**

Gene expression profiling analysis was performed to study the expression level of Ndrg1 during osteogenic and adipogenic differentiation. Gain-of-function and/or loss-of function experiments were carried out to study the role of NDRG1 in the proliferation and differentiation of marrow stromal progenitor cells and the mechanism underlying the function was investigated. Finally, in vivo transfection of Ndrg1 siRNA was done and its effect on osteogenic and adipogenic differentiation in mice was explored.

**Results:**

Gene expression profiling analysis revealed that NDRG1 level was regulated during osteogenic and adipogenic differentiation of progenitor cells. The functional experiments demonstrated that NDRG1 negatively regulated the cell growth, and reciprocally modulated the osteogenic and adipogenic commitment of marrow stromal progenitor cells, driving the cells to differentiate toward adipocytes at the expense of osteoblast differentiation. Moreover, NDRG1 interacted with low-density lipoprotein receptor-related protein 6 (LRP6) in the stromal progenitor cells and inactivated the canonical Wnt/β-catenin signaling cascade. Furthermore, the impaired differentiation of progenitor cells induced by Ndrg1 siRNA could be attenuated when β-catenin was simultaneously silenced. Finally, in vivo transfection of Ndrg1 siRNA to the marrow of mice prevented the inactivation of canonical Wnt signaling in the BMSCs of ovariectomized mice, and ameliorated the reduction of osteoblasts on the trabeculae and increase of fat accumulation in the marrow observed in the ovariectomized mice.

**Conclusion:**

This study has provided evidences that NDRG1 plays a role in reciprocally modulating osteogenic and adipogenic commitment of marrow stromal progenitor cells through inactivating canonical Wnt signaling.

**Supplementary Information:**

The online version contains supplementary material available at 10.1186/s13287-022-02714-5.

## Background

Bone marrow mesenchymal stem cells (BMSCs) have multi-directional differentiation potential, giving rise to osteoblasts, adipocytes, chondrocytes and myoblasts under different induction conditions. A reciprocal and competitive fate decision is frequently observed between adipogenesis and osteogenesis [1, 2]. Under normal physiological conditions, BMSCs have similar differentiation potential to osteoblasts and adipocytes. In certain pathological conditions, one aspect of osteogenesis or adipogenesis is dominant, which usually weakens the ability of BMSCs to differentiate in the other direction.

The reciprocal regulation of osteogenic and adipogenic differentiation of BMSCs is a finely orchestrated process, which involves a variety of transcription factors and complex signaling pathways. The best characterized regulators of osteogenesis include Wnt/β-catenin pathway, bone morphogenetic proteins (BMPs), runt related transcription factor-2 (Runx2), and osterix (OSX), etc. [3–7]. In contrast, peroxisome proliferator activated receptor γ (PPARγ) and CCAAT/enhancer binding proteins (C/EBPs) are the major transcription factors controlling adipocyte differentiation [8, 9].

N-myc downstream regulated gene 1 (NDRG1) is a member of NDRG family, which also includes NDRG2-4 [10]. Human NDRG1 contains 394 amino acids and is widely expressed in various tissues [10]. NDRG1 is a cytoplasmic protein and its C-terminal domain is unique compared to other NDRG members. Several serine and threonine sites at the C-terminus are the substrates for sequential phosphorylation by serum/glucocorticoid regulated kinase 1 (SGK1) and glycogen synthase kinase 3β (GSK3β) [10–12]. The phosphorylation of NDRG1 results in increased levels of the main adhesion molecules such as N-cadherin, α-catenin, and β-catenin [13].

NDRG1 is recognized as a regulator in a variety of biological processes such as cell growth, differentiation and adhesion, myelination, lipid synthesis, stress response and immune regulation [10]. Importantly, the role of NDRG1 in tumorigenesis and metastasis has been widely studied and controversial results suggest that NDRG1 may either suppress [14–19] or promote carcinogenesis and metastasis in cancers [20–23].

There are few studies reporting the regulation of bone cells and homeostasis by NDRG family members. Watari et al. have recently demonstrated the implication of NDRG1 in the differentiation of osteoclasts and bone remodeling [24]. A recent study reported the changed expression of NDRG1 in osteocytes of rats after treatment with sclerostin antibody [25]. However, the exact effect of NDRG1 on osteoblast differentiation has not been reported.

In the current study, we investigated whether and how NDRG1 regulates osteoblast differentiation. The data uncovered the anti-osteogenic and pro-adipogenic function of NDRG1 in mesenchymal progenitor cells. The mechanistic explorations revealed that the function of NDRG1 is based upon its inactivation of canonical Wnt signaling.

## Methods

### Cells

ST2 cells were maintained in α-MEM containing 10% FBS. Mouse BMSCs were isolated from the femurs and tibiae of 6 week-old C57BL/6 mice. Briefly, the marrow was flushed with α-MEM and the marrow cells were plated in α-MEM supplemented with 10% FBS. After 3 days of culturing, the non-adherent hematopoietic cells were removed and the culture medium was refreshed. The cells at passage 3–5 were induced to allow differentiation. For osteogenic differentiation, the cells at 70% confluence were cultured in osteogenic medium for 14 days followed by ALP staining, or for 21 days followed by alizarin red staining. For adipogenic differentiation, the cells at 100% confluence were cultured for 3 days in adipogenic medium, then for 2 days in presence of 5 μg/mL insulin. The osteogenic medium contains 50 μg/mL of ascorbic acid and 5 mmol/L of β-glycerophosphate in α-MEM supplemented with 10% FBS. The adipogenic medium contains 5 μg/mL insulin, 0.25 mM methylisobutylxanthine, 0.5 μM dexamethasone, and 50 μM indomethacin in α-MEM supplemented with 10% FBS.

### siRNAs, construct and transfections

For the loss-of-function studies, the siRNA targeting the CDS region of Ndrg1 or the control siRNA (Genepharma, Shanghai, China) was transfected for 24 h into cells using lipofectamine RNAi-max reagent (Invitrogen, Carlsbad, CA, USA) at the concentration of 20 nmol/L. For the gain-of-function studies, the CDS sequence of Ndrg1 cDNA was PCR-amplified by using specific primers and then cloned into pcDNA3.1(+) vector at BamHI/EcoRI restriction sites using the ClonExpress II One Step Cloning Kit (Vazyme, Nanjing, China). The resulting expression construct or the vector was transfected into cells in presence of the JetPRIME transfection reagent (Polyplus, Illkirch, France). After 4 h of transfection, the culture medium was refreshed. The sequences of the primers and siRNAs are listed in Additional file 1: Table S1 and S2, respectively.

### Lentiviral packaging and infection

For the loss-of-function experiments, the shRNA expression construct was made by inserting the annealed oilogo pair coding for the shRNA of Ndrg1 into the BamHI/EcoRI sites of pLVX-shRNA2 vector (Clontech, Mountain View, CA, USA). For the gain-of-function experiments, the overexpression construct was generated by subcloning the CDS sequence of Ndrg1 into the lentiviral vector pCDH-CMV-MCS-EF1-copGFP at the sites of EcoRI/BamHI. The lentiviral constructs or their vectors were transfected, respectively, into 293 T cells along with the packaging plasmids and the lentiviruses were packaged using a kit from Jiman Biotech (Shanghai, China). The primary BMSCs were infected with the viruses (multiplicity of infection = 20) to render the cells overexpressing or underexpressing Ndrg1.

### Cell proliferation assay

Cells were seeded in a 96-well plate at the density of 1.5 × 10^4^ per well. The cells were transfected with Ndrg1 expression construct or the vector for 4 h in presence of the JetPRIME transfection reagent. The effect of NDRG1 on cell growth was determined by using a CCK-8 cell counting kit (Vazyme, Nanjing, China).

### Protein stability assay

ST2 cells transfected with Ndrg1 expression construct or the vector were treated with cycloheximide (100 μg/ml). Cell lysates were collected at the indicated time points and run on SDS-PAGE following protein concentration assay with a BCA kit (Beyotime, Shanghai, China). The amount of low-density lipoprotein receptor-related protein 6 (LRP6) was detected by Western blotting.

### Quantitative reverse transcription (RT)-PCR

Total RNA was extracted from cells using a RNA extraction kit (Omega, USA), or from tissues using RNAiso Plus (Takara, Dalian, China). Then, the RNA was reverse transcribed using RevertAid First Strand cDNA Synthesis Kit (Thermo Fisher, Waltham, MA, USA). The resulting cDNAs were used as templates for PCR using the SYBR-Green Master PCR Mix (Sangon Biotech, Shanghai, China). The relative quantitation value for each target gene was calculated following the comparative Ct method using β-actin as the reference gene. The primers for quantitative PCR are listed in Additional file 1: Table S1.

### Western blot analysis

Protein extracts were run on 10–15% SDS–polyacrylamide gel electrophoresis (PAGE) and transferred to nitrocellulose membranes. After blocking with 5% skim milk, the membranes were probed with primary antibodies, afterwards incubated with horseradish peroxidase-conjugated secondary antibodies. Finally, the bands were visualized using a chemiluminescence reagent (Proteintech, Wuhan, China). The primary antibodies included antibodies from Cell Signaling Technology (Danvers, MA, USA): anti-Runx2 (#12,556), anti-phospho-LRP6 (Ser1490) (#2568), anti-non-phospho-β-catenin (#8814), anti-C/EBPα (#8178), and anti-PPARγ (#2443); antibodies from Abcam (Cambridge, MA, USA): anti-NDRG1 (ab124689), anti-osterix (ab94744), anti-LRP6 (ab134146), anti-transcription factor 7 like 2 (TCF7L2) (ab76151), and anti-alkaline phosphatase (ALP) (ab108337); antibodies from Proteintech (Wuhan, China): anti-fatty acid binding protein 4 (FABP4) (12802-1-AP), anti-osteopontin (25715-1-AP), anti-β-catenin (51067-2-AP), and anti-β-actin (66009-1-Ig).

### ALP staining and alizarin red staining

For ALP staining, the differentiated osteoblasts following 14 days of osteogenic induction were rinsed with PBS, fixed with 4% paraformaldehyde for 10 min, then stained with NBT/BCIP staining solution (Beyotime, Shanghai, China) for 15 min. For alizarin red staining, the cultures were fixed with 4% paraformaldehyde for 10 min, washed with deionized water, and stained with 1% alizarin red stain solution (pH 4.2) for 5 min.

### Oil-red O staining

Following adipogenic induction, the differentiated cultures were fixed with 4% paraformaldehyde, then rinsed with water and washed with 60% saturated isopropanol. Subsequently, the samples were stained with 0.3% oil-red O (w/v, dissolved in 60% saturated isopropanol) for 5 min. After observation under the microscope, the retention of the dye was determined by measuring the spectrophotometry at 520 nm.

### Co-immunoprecipitation

The supernatant of centrifuged cell lysate was incubated with anti-NDRG1 (final concentration: 1 μg/ml) at 4 °C overnight, followed by incubating with protein A/G magnetic beads for 2 h at 4 °C. The beads were washed with PBS + 0.5% Triton X100, then boiled for 5 min in SDS sample buffer. The IP eluates were run on 10% SDS-PAGE gel, transferred to a nitrocellulose membrane, and probed with anti-LRP6.

### Cellular immunofluorescence

Cellular immunofluorescence staining for β-catenin was performed. Briefly, the ST2 cells overexpressing or underexpressing Ndrg1, or their negative control cells were fixed with 4% paraformaldehyde, permeabilized with 1% Triton X-100, and then blocked in 1% bovine serum albumin (BSA). The cells were then probed overnight with anti-β-catenin antibody (Proteintech, Wuhan, China) at 4 ºC. After a wash with PBS, the cells were incubated with Alexa Fluor 488-conjugated IgG (Proteintech, Wuhan, China) to detect the bound proteins. The cells were finally counterstained with DAPI and observed under a fluorescence microscope.

### In vivo delivery of siRNA

C57BL/6 mice were purchased from SPF Biotechnology (Beijing, China) and housed in SPF facility with a 12 h:12 h light/dark cycle and free access to food and water. The in vivo efficacy of the Ndrg1 siRNA was tested in mice 3 days after receiving intra-tibial transfection of 10 μg 2’-Ome modified Ndrg1 siRNA or control siRNA (Genepharma, Shanghai, China). The in vivo transfection procedure was described previously [26]. Briefly, for one mouse, 10 μg 2’-Ome modified siRNA (Genepharma, Shanghai, China) and 1.5 μL in vivo-jetPEI (Polyplus, Illkirch, France), each diluted with 12.5 μl of 5% glucose, were mixed by vortexing. Then, the complexed siRNA was delivered to the marrow of the mouse tibiae. BMSCs were flushed from the tibiae, grown in α-MEM containing 10% FBS, subjected to RNA extraction and thereafter qRT-PCR. To examine the in vivo role of NDRG1, the female mice aged 8 weeks were randomly allocated into four groups: Sham/Ctrl siRNA group (sham-operated mice receiving control siRNA), OVX/Ctrl siRNA group (ovariectomized mice receiving control siRNA), Sham/Ndrg1 siRNA group (sham-operated mice receiving Ndrg1 siRNA) and OVX/Ndrg1 siRNA group (ovariectomized mice receiving Ndrg1 siRNA). The mice received ovariectomy or sham surgery 7 days after the first transfection, and received additional transfections every 3 weeks after the first transfection. 4 weeks after the surgery, some mice were sacrificed and BMSCs were isolated from the transfected tibiae and cultured in α-MEM containing 10% FBS. The cells at passage 3 were subjected to protein extraction and Western blotting analysis of Wnt/β-catenin pathway proteins. Additionally, the cells at passage 3 were induced to allow osteogenic or adipogenic differentiation. The other mice were sacrificed 12 weeks after the first transfection and the tibiae were collected.

### Histological and immunohistochemical staining

Tibiae samples were fixed in 10% neutral buffered formalin, decalcified in 14% EDTA (pH 7.4), and embedded in paraffin. 5 μm thick sagittal-oriented sections were prepared and then stained with hematoxylin and eosin (H&E). The quantity of the bone marrow fat was measured by calculating the numbers and areas of adipocytes in the sections.

For the examination of osteocalcin-positive osteoblasts, immunohistochemical staining was done on 5-µm paraffin-embedded tissue sections. After deparaffinization and rehydration, antigen retrieval was carried out by digesting the sections with 0.05% trypsin at 37 °C for 15 min, followed by incubation with hydrogen peroxide to quench endogenous peroxidases. After blocking with 1% BSA, the sections were incubated at 37 °C with primary anti-osteocalcin antibody (Proteintech, Wuhan, China) for 2 h, and then incubated with HRP-conjugated secondary antibody. 3,3’-diaminobenzidine (DAB) was employed to visualize the osteocalcin-positive osteoblasts. Nuclei were counterstained with hematoxylin. The region of interest starts from 0.1 mm below the growth plate and is 1 mm in length.

### Statistical analysis

Data are presented as mean ± SD. SPSS was employed to conduct statistical analyses. Student’s t test was done for comparisons between two groups. For comparisons among 3 or more groups, one-way ANOVA test was employed for those with only one independent variable and two-way ANOVA was done for those with two independent variables. The post-hoc comparisons were done using Tukey test. A value of *p* < 0.05 was regarded to be significant.

## Results

### Ndrg1 was regulated during differentiation of marrow stromal progenitor cells

**Fig. 1:**
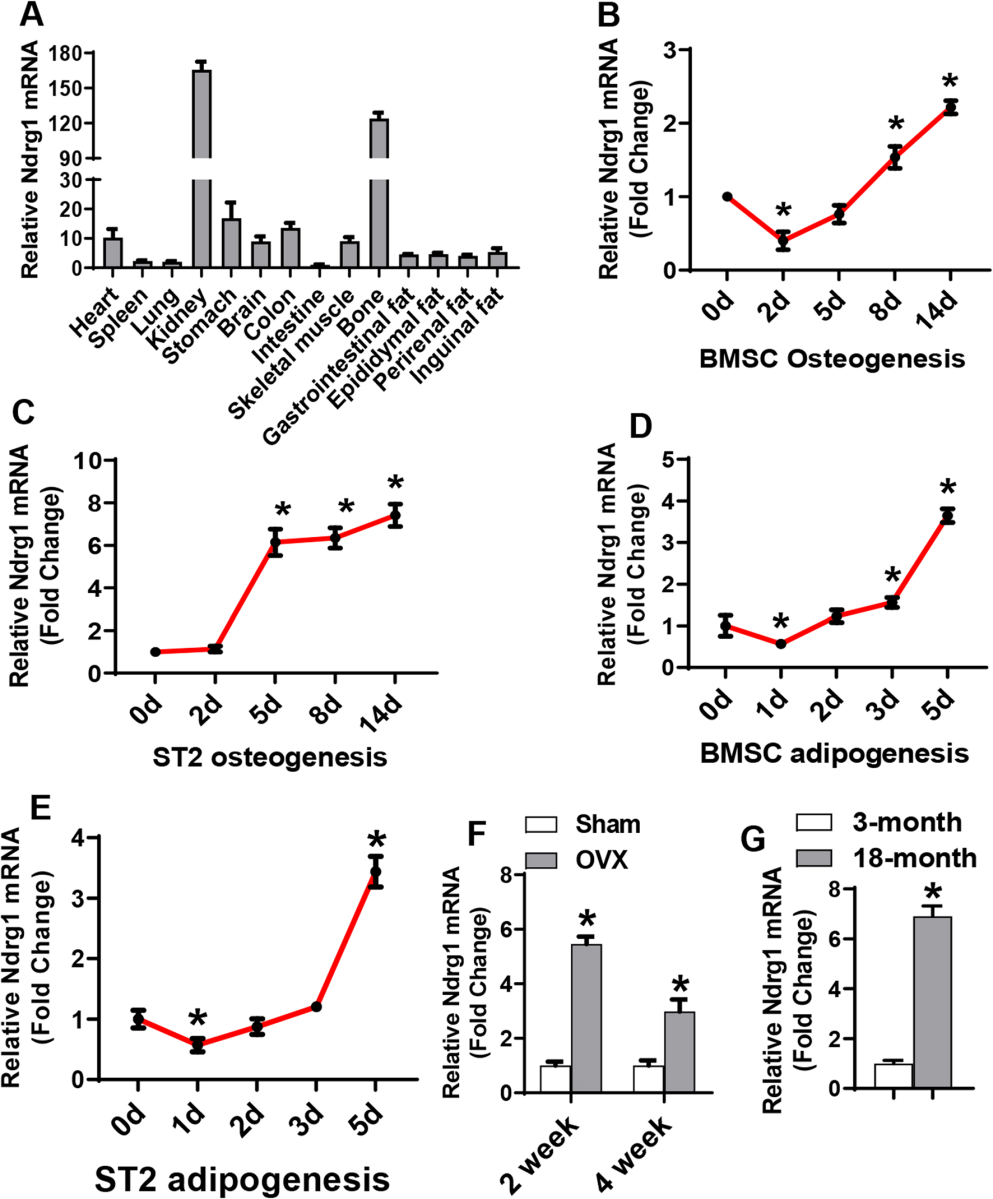
Ndrg1 was regulated during differentiation of marrow stromal progenitor cells. Ndrg1 mRNA expression was detected in various tissues of mice using qRT-PCR (**A**). The level of Ndrg1 in intestine was set at 1. Ndrg1 expression was assayed using qRT-PCR in primary BMSCs and ST2 cells at indicated time points during osteogenesis or adipogenesis (**B–E**). Ndrg1 expression was assayed in the BMSCs of ovariectomized mice 2 weeks or 4 weeks after surgery (**F**) or in the calvaria of 18-month-old mice (**G**). Values represent mean ± SD, *n* = 3. *Significant versus day 0 (**B–E**) or Sham (**F**) or 3-month-old mice (**G**), *p* < 0.05

The expression level of Ndrg1 was investigated in various tissues of 6-week-old mice. qRT-PCR revealed the abundance of Ndrg1 mRNA in kidney and bone (Fig. 1A). Ndrg1 was moderately expressed in heart, brain, colon, skeletal muscle and various adipose tissues (Fig. 1A). It was shown by using qRT-PCR that the expression of Ndrg1 was downregulated at day 2, but upregulated at day 8 through 14 in primary BMSCs during osteogenic differentiation (Fig. 1B). For ST2 cells, Ndrg1 was upregulated at day 5 through day 14 (Fig. 1C). By contrast, Ndrg1 was downregulated at the early stage (day 1) and upregulated at later stage (day 3 and/or day 5) of induction during adipogenic differentiation (Fig. 1D, E). Moreover, mRNA expression assay revealed that Ndrg1 was increased in the BMSCs from ovariectomized (OVX) mice versus those from sham-operated mice (Fig. 1F) or in the calvaria from 18-month-old aged mice versus 3-month-old young mice (Fig. 1G). These data suggest that NDRG1 may play a role in osteogenic and/or adipogenic differentiation of progenitor cells.

### NDRG1 suppressed osteogenic differentiation of stromal progenitor cells

**Fig. 2:**
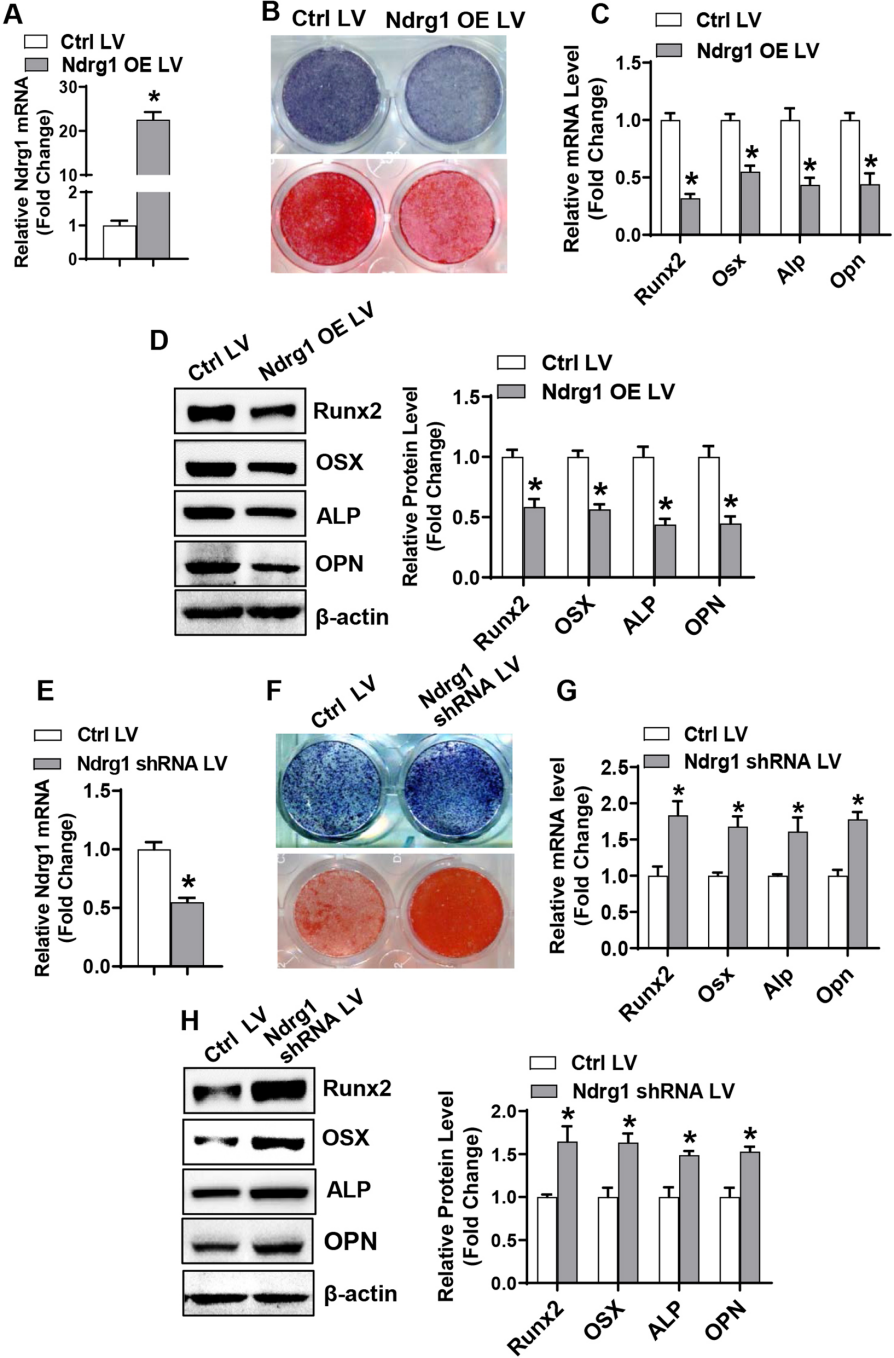
NDRG1 suppressed osteogenic differentiation of primary BMSCs. Overexpression (**A**) or silencing (**E**) of Ndrg1 in primary BMSCs after infection of Ndrg1 expression lentivirus or shRNA expression lentivirus was verified using qRT-PCR. Primary BMSCs overexpressing or underexpressing Ndrg1 were induced to allow osteogenic differentiation. The effects of Ndrg1 overexpression (**B–D**) or silencing (**F–H**) on osteogenic differentiation were examined. ALP staining and alizarin red staining were performed in differentiated osteoblasts 14 days and 21 days, respectively, after osteogenic treatment (**B**, **F**). The mRNA (**C**, **G**) and protein (**D**, **H**) levels of osteogenic factors were measured 72 h after osteogenic treatment. Values represent mean ± SD, *n* = 3. *Significant versus control LV, *p* < 0.05

Gain-of-function and loss-of-function experiments were done to study the role of NDRG1 in osteogenesis. For the gain-of-function study, qRT-PCR analysis revealed the successful overexpression of Ndrg1 in primary BMSCs following the infection of the Ndrg1 expression lentivirus (Fig. 2A). Following osteogenic treatment, overexpression of Ndrg1 blocked osteogenic differentiation, as indicated by the attenuated alkaline phosphatase (ALP) staining and alizarin red staining (Fig. 2B), and the decreased mRNA and protein levels of the osteogenic factors such as Runx2, osterix, ALP and osteopontin as compared to the infection of the control virus (mRNAs decreased by 45–68% and proteins decreased by 42–56%) (Fig. 2C, D). Moreover, Ndrg1 overexpression also alleviated osteogenic differentiation of ST2 cells (Additional file 2: Fig. S1A–D).

For the loss-of-function study, qRT-PCR analysis revealed the successful downregulation of Ndrg1 after the infection of the Ndrg1 shRNA lentivirus in primary BMSCs (Fig. 2E). Following osteogenic treatment, knockdown of Ndrg1 stimulated osteogenic differentiation, as indicated by the enhanced ALP staining and alizarin red staining (Fig. 2F), and the increased mRNA and protein levels of the osteogenic factors as compared to the infection of the control virus (mRNAs increased by 61–83% and proteins increased by 49–65%) (Fig. 2G, H). Moreover, Ndrg1 depletion also promoted osteogenic differentiation of ST2 cells (Additional file 2: Fig. S1E–H). Furthermore, Ndrg1 overexpression suppressed the cell growth rate of ST2 cells by 17% (Fig. S1I).

### NDRG1 stimulated adipogenic differentiation of stromal progenitor cells

**Fig. 3:**
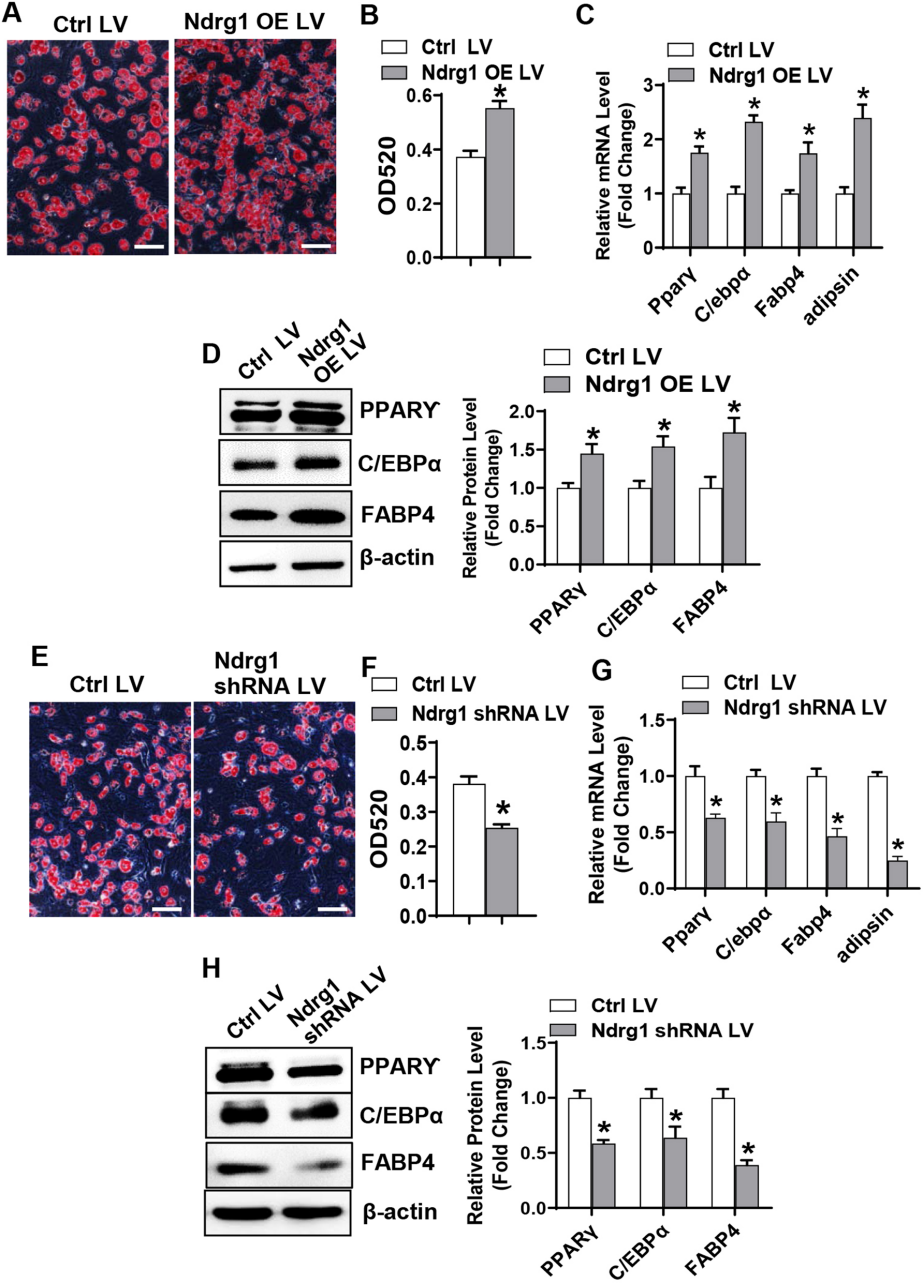
NDRG1 stimulated adipogenic differentiation of primary BMSCs. Primary BMSCs infected with Ndrg1 overexpression or shRNA lentivirus were induced to allow adipogenic differentiation. The effects of Ndrg1 overexpression (**A–D**) or silencing (**E–H**) on adipogenic differentiation were examined. Oil-red O staining was performed in differentiated adipocytes 5 days after adipogenic treatment (**A**, **E**) and the stain extracted with isopropanol was measured at 520 nm by spectrophotometry (**B**, **F**). The mRNA (**C**, **G**) and protein (**D**, **H**) levels of adipogenic factors were measured 48 h and 72 h, respectively, after adipogenic treatment. Image scale in **A**, **E**: 100 μm. Values represent mean ± SD, *n* = 3. *Significant versus control LV, *p* < 0.05

For the gain-of-function experiments, after adipogenic treatment, overexpressing Ndrg1 in primary BMSCs stimulated adipogenic differentiation, which was evidenced by the enhanced oil-red O staining (49% increase of oil-red O retention in the cells) (Fig. 3A, B) and the induced mRNA and protein levels of the adipogenic factors such as PPARγ, C/EBPα, FABP4 and adipsin as compared to the infection of the control virus (mRNAs increased by 1.7–2.4 fold and proteins increased by 45–72%) (Fig. 3C, D). Additionally, overexpressing Ndrg1 in ST2 cells promoted the adipogenic differentiation as well (Additional file 2: Fig. S2A–D).

For the loss-of-function experiments, after adipogenic treatment, downregulating Ndrg1 in primary BMSCs blunted adipogenic differentiation, which was evidenced by the attenuated oil-red O staining (33% decrease of oil-red O retention in the cells) (Fig. 3E, F) and the decreased mRNA and protein levels of the adipogenic factors as compared to the infection of the control virus (mRNAs decreased by 37–75% and proteins decreased by 36–61%) (Fig. 3G, H). Additionally, downregulating Ndrg1 in ST2 cells blunted the adipogenic differentiation as well (Additional file 2: Fig. S2E–H).

### NDRG1 interacted with LRP6 and inactivated canonical Wnt signaling

**Fig. 4:**
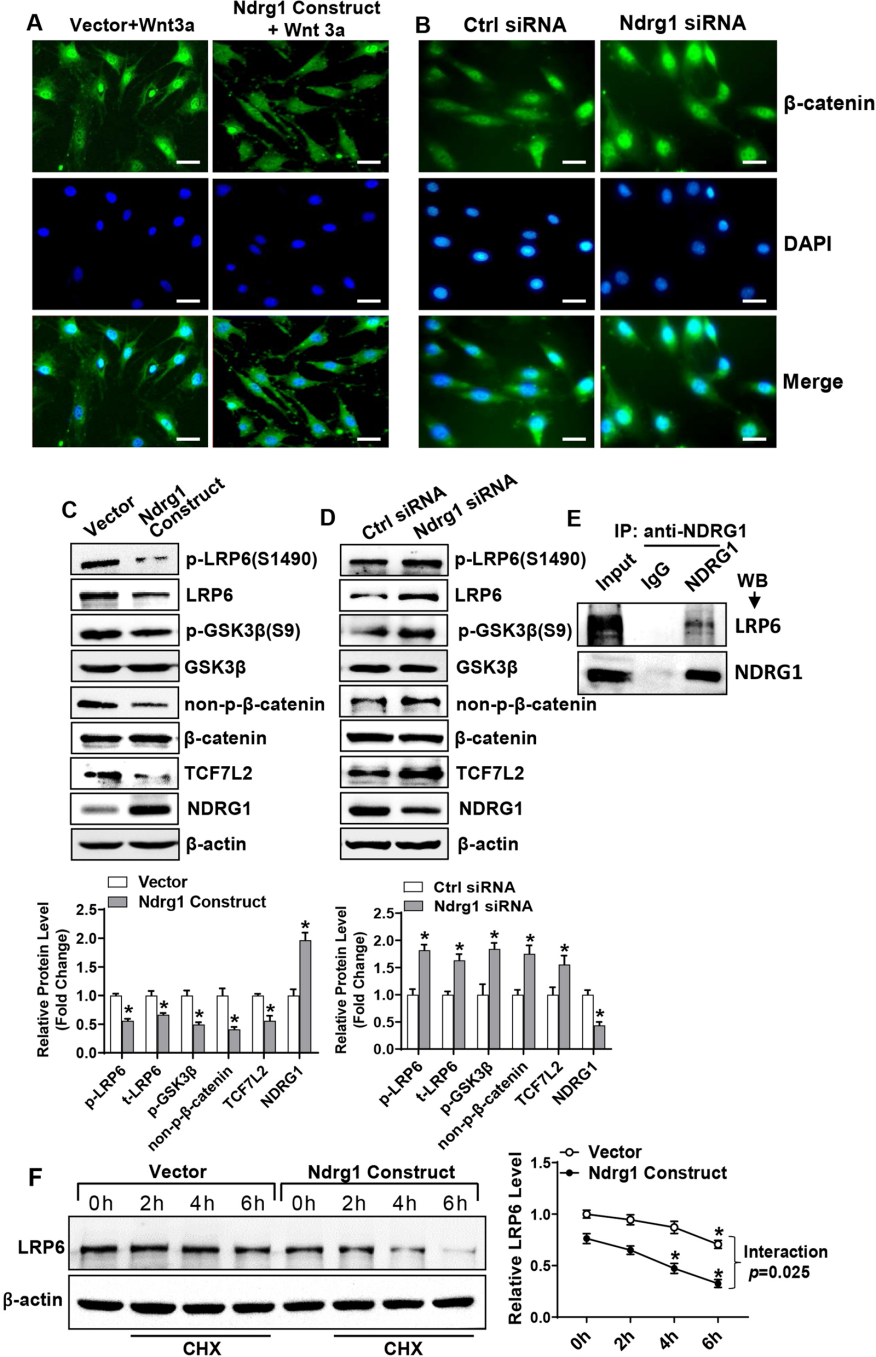
NDRG1 interacted with LRP6 and inactivated canonical Wnt signaling. The nuclear translocation of β-catenin was examined in ST2 cells transfected with Ndrg1 expression construct (**A**) or siRNA (**B**). The protein levels of the major components of canonical Wnt signaling were measured using Western blotting in undifferentiated ST2 72 h after overexpression of Ndrg1 (**C**), or silencing of Ndrg1 (**D**). Co-IP was done to detect the interaction between NDRG1 and LRP6 (**E**). ST2 cells transfected with Ndrg1 expression construct or the vector were treated with cycloheximide and the amount of LRP6 was detected by Western blotting (**F**). Image scale in **A**, **B**: 50 μm. Values represent mean ± SD, *n* = 3. *Significant versus vector (**C**), or control siRNA (**D**), or 0 h (**F**), *p* < 0.05

To explore the mechanisms for the regulation of progenitor cell differentiation, cellular immunofluorescence staining of β-catenin was carried out in ST2 cells. It revealed that the dysregulation of NDRG1 changed the intracellular distribution of β-catenin. Briefly, Ndrg1 overexpression inhibited, while Ndrg1 depletion promoted the translocation of β-catenin from the cytoplasm into the nucleus (Fig. 4A, B). Consistent to this, Western blotting analysis showed that the overexpression of NDRG1 significantly reduced the protein levels of phospho-LRP6(S1490), phospho-GSK3β(S9), non-phospho-β-catenin and transcription factor 7 like 2 (TCF7L2). Conversely, these proteins were increased following depletion of Ndrg1 (Fig. 4C, D). Furthermore, the result from Co-IP experiment showed that NDRG1 physically interacted with LRP6 protein (Fig. 4E).

Additionally, the stability of LRP6 was determined in the cells overexpressing NDRG1 following treatment with cycloheximide to block protein synthesis. The results showed that LRP6 was more unstable in the cells overexpressing NDRG1 than in the cells transfected with vector (Fig. 4F).

### Silencing of β-catenin attenuated the impaired differentiation of marrow stromal cells induced by Ndrg1 siRNA

**Fig. 5:**
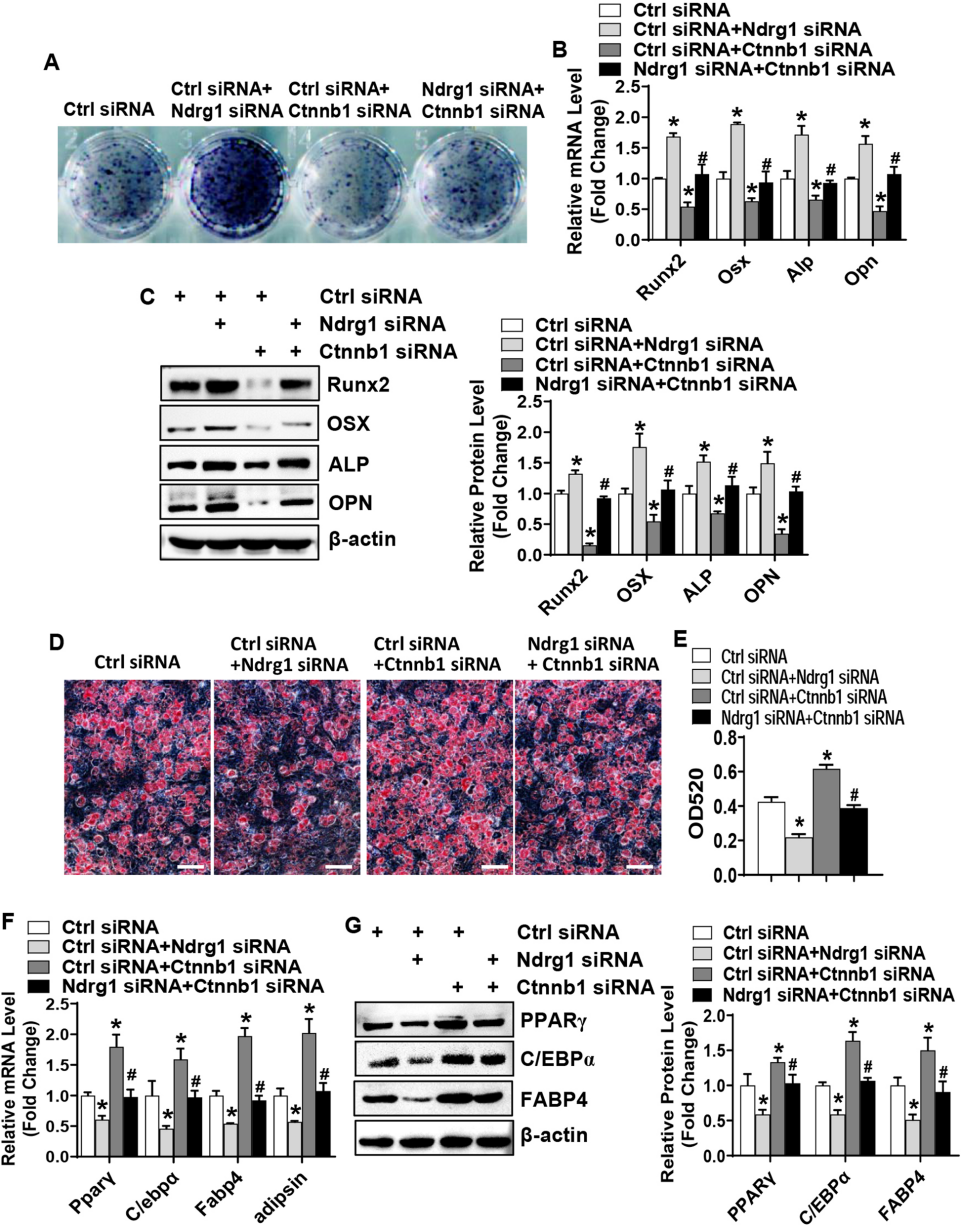
Silencing of β-catenin attenuated the pro-osteogenic and anti-adipogenic effects of Ndrg1 siRNA. ALP staining was performed in differentiated osteoblasts 14 days after osteogenic treatment of ST2 (**A**). The mRNA (**B**) and protein (**C**) levels of osteogenic factors were measured 72 h after osteogenic treatment. Oil-red O staining was performed in differentiated adipocytes 5 days after adipogenic treatment of ST2 (**D**) and the stain extracted with isopropanol was measured at 520 nm by spectrophotometry (**E**). The mRNA (**F**) and protein (**G**) levels of adipogenic factors were measured 48 h and 72 h, respectively, after adipogenic treatment. Image scale in **D**: 100 μm. Values represent mean ± SD, *n* = 3. *Significant versus control siRNA, *p* < 0.05. ^#^Significant versus control siRNA plus Ndrg1 siRNA

To further validate the involvement of canonical Wnt signaling in the effect of Ndrg1 siRNA, we performed cotransfection experiments. Under osteogenic condition, the cells cotransfected with Ndrg1 siRNA and control siRNA displayed enhanced ALP staining and increased levels of osteogenic factors as compared to the cells transfected with control siRNA only. This pro-osteogenic effect of Ndrg1 siRNA was attenuated when the cells were cotransfected with Ndrg1 siRNA and β-catenin siRNA (Fig. 5A–C). By contrast, under adipogenic condition, the cells cotransfected with Ndrg1 siRNA and control siRNA displayed alleviated oil-red O staining and decreased levels of adipogenic factors as compared to the cells transfected with control siRNA only. This anti-adipogenic effect of Ndrg1 siRNA was attenuated when the cells were cotransfected with Ndrg1 siRNA and β-catenin siRNA (Fig. 5D–F).

### Silencing of Ndrg1 prevented the inactivation of canonical Wnt signaling and ameliorated the impaired differentiation of BMSCs in ovariectomized mice

**Fig. 6:**
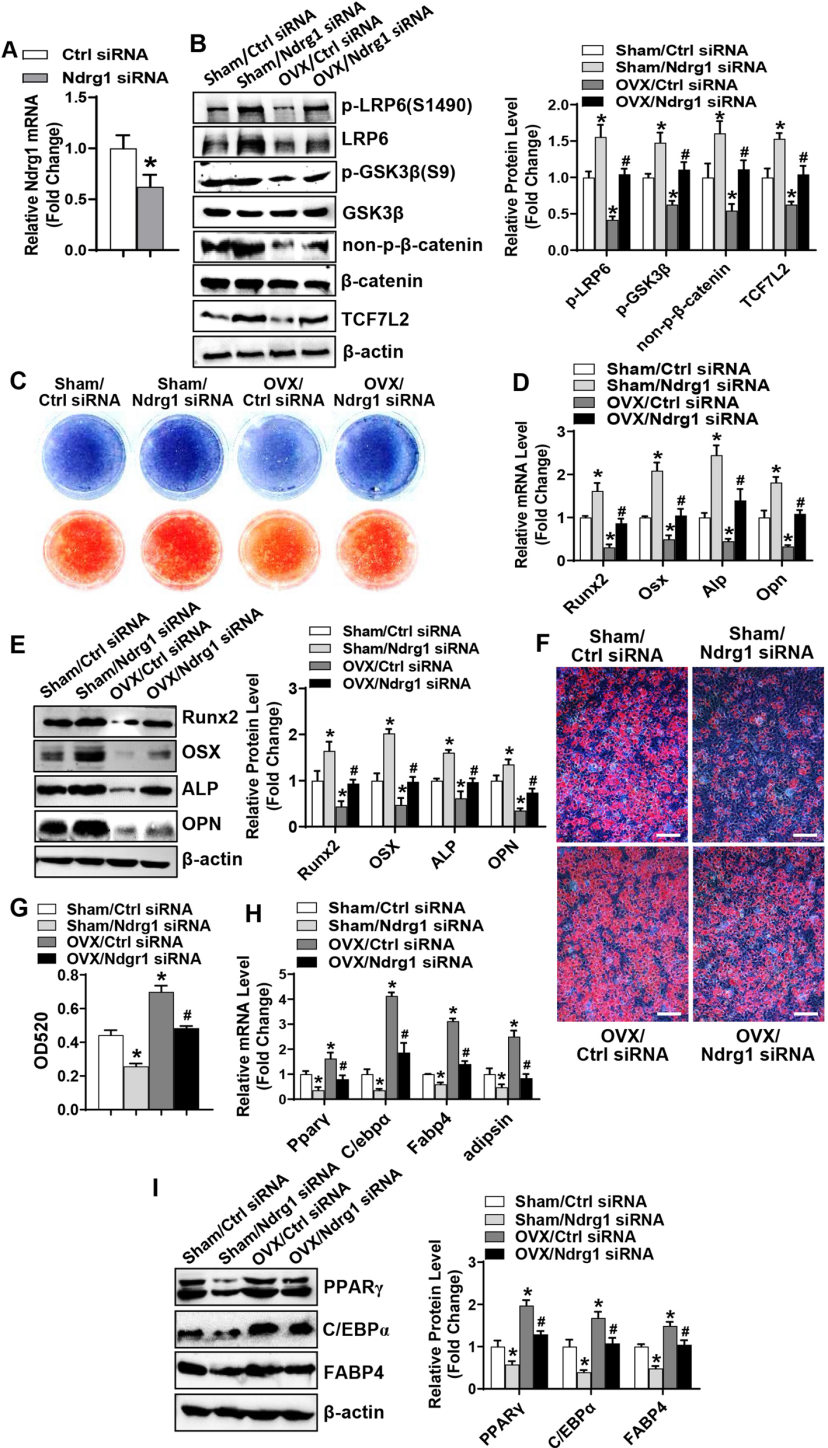
Silencing of Ndrg1 prevented the inactivation of canonical Wnt signaling and ameliorated the impaired differentiation of BMSCs from ovariectomized mice. qRT-PCR was done to verify the downregulation of Ndrg1 mRNA in BMSCs depleted of Ndrg1 (**A**). BMSCs were isolated and cultured, and proteins were extracted and subjected to Western blotting (**B**). BMSCs were induced to allow osteogenic (**C**–**E**) or adipogenic differentiation (**F**–**I**). ALP staining and alizarin red staining were performed in differentiated osteoblasts 14 days and 21 days, respectively, after osteogenic treatment (**C**). The mRNA (**D**) and protein (**E**) levels of osteogenic factors were measured 72 h after osteogenic treatment. Oil-red O staining was performed in differentiated adipocytes 5 days after adipogenic treatment (**F**) and the stain extracted with isopropanol was measured at 520 nm by spectrophotometry (**G**). The mRNA (**H**) and protein (**I**) levels of adipogenic factors were measured 48 h and 72 h, respectively, after adipogenic treatment. Image scale in **F**: 100 μm. Data are mean ± SD, *n* = 3. *p < 0.05 versus Sham/Ctrl siRNA. ^#^*p* < 0.05 versus OVX/Ctrl siRNA

We further explored the physiological role of NDRG1 through investigating the effect of Ndrg1 siRNA on the differentiation of BMSCs in mice. qRT-PCR analysis validated the efficacy of Ndrg1 siRNA in knocking down the expression of endogenous Ndrg1 in the BMSCs of tibiae (Fig. 6A). 4 weeks after the surgery, BMSCs were isolated and cultured. Western blotting analysis revealed that compared to the Sham/Ctrl siRNA group, the phospho-LRP6(S1490), phospho-GSK3β(S9), non-phospho-β-catenin and TCF7L2 were increased in the Sham/Ndrg1 siRNA group and decreased in the OVX/Ctrl siRNA group. By contrast, these proteins were increased in the OVX/Ndrg1 siRNA group as compared to the OVX/Ctrl siRNA group (Fig. 6B).

Furthermore, we investigated the osteogenic and adipogenic capability of the BMSCs (Fig. 6C–I). Following osteogenic induction, osteogenic differentiation was induced in Sham/Ndrg1 siRNA group, whereas it was attenuated in OVX/Ctrl siRNA group as compared to Sham/Ctrl siRNA group. The OVX/Ndrg1 siRNA group showed enhanced osteogenic differentiation versus OVX/Ctrl siRNA group (Fig. 6C–E). Conversely, following adipogenic induction, adipogenic differentiation was repressed in Sham/Ndrg1 siRNA group, whereas it was induced in OVX/Ctrl siRNA group as compared to Sham/Ctrl siRNA group. The OVX/Ndrg1 siRNA group showed repressed adipogenic differentiation versus OVX/Ctrl siRNA group (Fig. 6F–I).

**Fig. 7:**
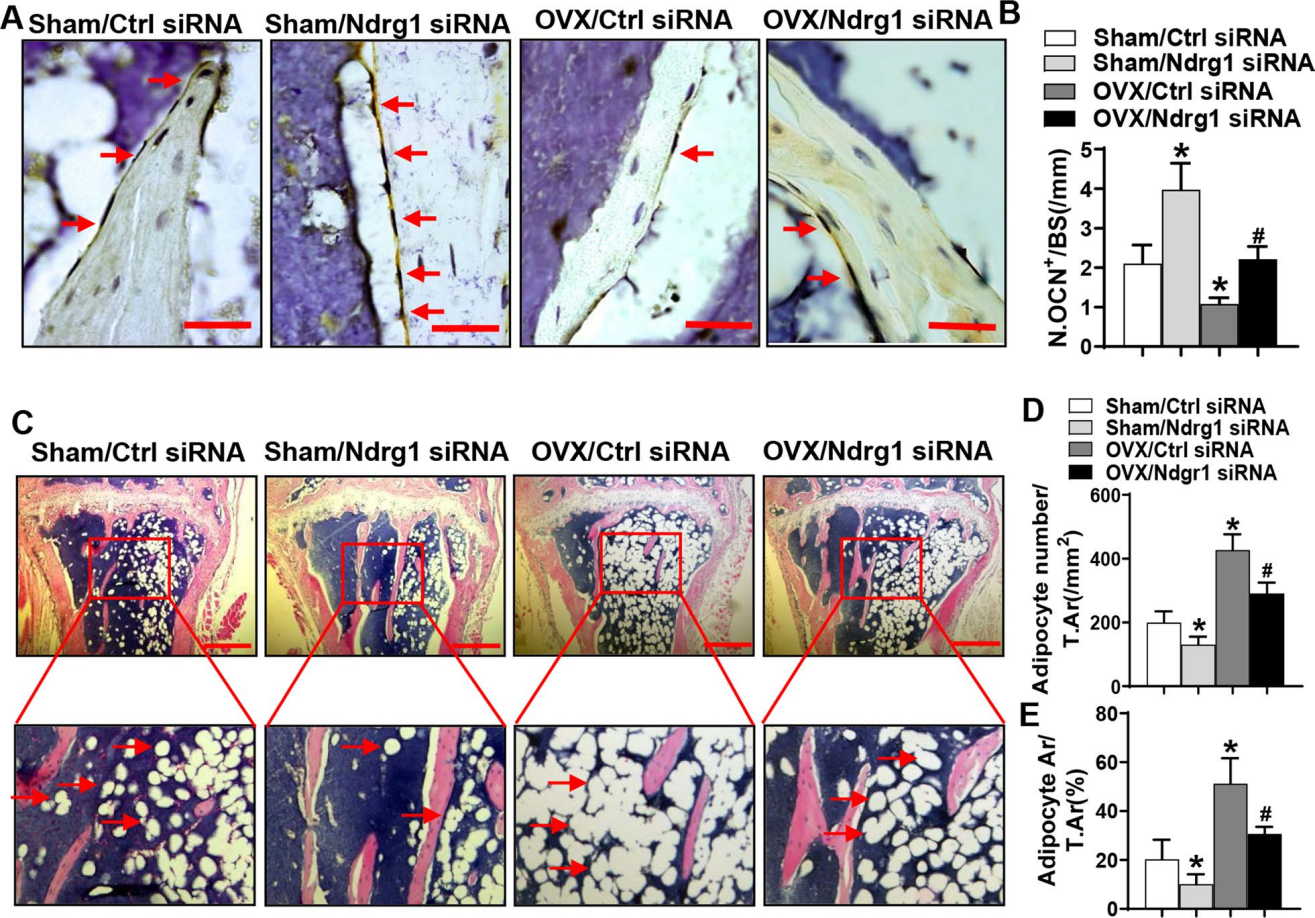
Silencing of Ndrg1 increased osteoblasts and ameliorated marrow adiposity in ovariectomized mice. Representative images of osteocalcin immunohistochemical staining are shown. Image scale: 25 μm (**A**). Numbers of osteoblasts on the trabecular bone surfaces in the metaphysis were counted (**B**). H&E staining was done. Image scale, 400 μm (**C**). Numbers (**D**) and areas (**E**) of adipocytes were quantified. Data are mean ± SD. *n* = 6. *p < 0.05 versus Sham/Ctrl siRNA. ^#^*p* < 0.05 versus OVX/Ctrl siRNA

Moreover, 12 weeks after the first transfection, the osteocalcin-positive osteoblasts on the trabeculae were increased in the Sham/Ndrg1 siRNA group and were decreased in the OVX/Ctrl siRNA group as compared to the Sham/Ctrl siRNA group. The OVX/Ndrg1 siRNA group had more osteoblasts versus the OVX/Ctrl siRNA group (Fig. 7A, B). By contrast, the number and the area of adipocytes in the marrow were decreased in the Sham/Ndrg1 siRNA group and were increased in the OVX/Ctrl siRNA group as compared to the Sham/Ctrl siRNA group. The OVX/Ndrg1 siRNA group had fewer adipocytes versus the OVX/Ctrl siRNA group (Fig. 7C–E).

## Discussion

There has been an increasing interest concerning the function of NDRG1 in regulating cell differentiation. In addition to its required role in the formation of adipocytes from precursor cells [27], NDRG1 has recently been implicated in the differentiation of osteoclasts and bone remodeling [24]. NDRG1 gene knockout mice exhibited abnormal spinal curvature, increased trabecular bone mass and decreased number of osteoclasts. The differentiation capacity of bone marrow cells into mature osteoclasts was downregulated in the mutant mice following stimulation with macrophage colony stimulating factor (M-CSF) and receptor activator of NF-κB ligand (RANKL) [24]. However, it is not known if and how NDRG1 plays a role in the cell fate decision of BMSCs.

In the current study, we explored the function of NDRG1 in regulating the osteogenic differentiation of progenitor cells. The expression profiling analysis revealed that NDRG1 was highly expressed in bone. Moreover, the expression of NDRG1 was regulated during either osteogenic or adipogenic differentiation of marrow stromal progenitor cells. These data suggest the potential role of NDRG1 as a regulator of the differentiation of progenitor cells.

We then unearthed the exact function of NDRG1 by employing gain-of-function and loss-of function studies. Similar to its inhibitory effect on tumorigenesis, NDRG1 negatively regulated the cell growth of stromal progenitor cells. Moreover, NDRG1 reciprocally modulated the osteogenic and adipogenic differentiation of marrow stromal progenitor cells, driving the cells to differentiate toward adipocytes at the expense of osteoblast differentiation. Of more importance, the physiological role of NDRG1 was validated as the downregulation of NDRG1 in the marrow of mice was able to increase osteoblasts and decrease adipocytes in either the wild-type mice or the ovariectomized mice. Of interest, this effect is opposite to the role of NDRG2, which was shown to stimulate in vitro osteoblast differentiation through the JAK3/STAT3 signal pathway [28].

Interestingly, it has recently been documented that the depletion of Ndrg1 suppressed the production of osteocalcin and enhanced cell proliferation, along with the suppression of p21 expression in osteosarcoma cells, suggesting the potential role of Ndrg1 in the cell differentiation of osteosarcoma cells [29]. Thus, NDRG1 may play completely different roles in the differentiation of osteosarcoma and non-neoplastic osteoblasts.

Canonical Wnt signaling is among the best characterized signaling pathways that are critical in modulating the development and homeostasis of bone. The activation of the canonical Wnt signaling usually increases, while the inhibition of the pathway decreases bone mass and strength [30]. Canonical Wnt signaling is involved as a major mechanism in the control of osteogenesis and bone mass accrual by many regulators such as BMPs, nuclear factor-I (NFI) family members and microRNAs [31–36]. In the current study, we explored if the canonical Wnt/β-catenin signaling is involved in the anti-osteogenic and pro-adipogenic activity of NDRG1. In line with our hypothesis, NDRG1 suppressed the translocation of β-catenin from the cytoplasm to the nucleus, and downregulated the protein levels of active non-phospho-β-catenin and TCF7L2, demonstrating its inhibitory effect on canonical Wnt pathway. Of importance, the downregulation of NDRG1 in the marrow of mice was able to activate the canonical Wnt signaling in either the wild-type mice or the ovariectomized mice.

It is commonly known that LRP6, one of the co-receptors of Wnt ligands, is required for the transduction of Wnt/β-catenin signaling cascade. Upon stimulation with Wnt, the Frizzled/LRP6 complex recruits Disheveled-1 polymers which, in turn, recruit the AXIN1/GSK3β complex to the plasma membrane, facilitating the formation of signalosomes and blocking AXIN1/GSK3-mediated phosphorylation and subsequent degradation of β-catenin [37]. A key step in this procedure is the Wnt-induced phosphorylation of of LRP6 at five reiterated PPPSPxS motifs and adjacent Ser/Thr clusters [38]. It was reported that NDRG1 is capable of interacting with LRP6 and preventing its phosphorylation by Wnt ligands, thereby blocking de-phosphorylated inactivation of GSK3β and subsequent activation of Wnt signaling [16]. Consistent to this, we also demonstrated that NDRG1 directly interacted with LRP6 and reduced the phosphorylation of LRP6 in stromal cells. Of interest, in our study, NDRG1 also decreased total LRP6. To further clarify the mechanism, we investigated the stability of LRP6 by using cycloheximide. It was shown that NDRG1 overexpression rendered LRP6 more unstable. Collectively, these data suggest that the inhibition of Wnt/β-catenin signaling cascade by NDRG1 is mediated through its binding and facilitating the inactivation and degradation of LRP6.

We further tried to elucidate if canonical Wnt signaling is implicated in the modulation of progenitor cell differentiation by NDRG1. NDRG1 loss-of-function study was carried out under the background of β-catenin silencing. The pro-osteogenic and anti-adipogenic effects of Ndrg1 siRNA were attenuated when the cells were depleted of β-catenin. This supports the hypothesis that NDRG1 suppresses osteogenesis and conversely promotes adipogenesis via interacting with LRP6 to inactivate Wnt/β-catenin signaling cascade.

## Conclusion

In summary, the current study has provided evidences that NDRG1 plays a role in cell fate decision of progenitor cells, favoring adipocyte formation at the expense of osteoblast differentiation in vitro and in vivo. Mechanistic investigations suggest that NDRG1 interacts with the Wnt co-receptor, LRP6, thereby blocks the canonical Wnt signaling, and thus orchestrates a cellular network that impairs the differentiation of marrow stromal progenitor cells. Taken as a whole, this study suggests that NDRG1 is an attractive option for the therapeutic intervention of metabolic disorders such as osteoporosis.

## Supplementary Information

**Additional file 1**. Supplementary tables 1 and 2.

**Additional file 2**. Supplementary figures 1 and 2.

## Data Availability

The authors confirm that all data underlying the findings are fully available.

## Abbreviations

ALP: Alkaline phosphatase
BMP2: Bone morphogenetic protein 2
BMSCs: Bone marrow mesenchymal stem cells
C/EBP: CCAAT/enhancer binding protein
FABP4: Fatty acid binding protein 4
GSK3β: Glycogen synthase kinase-3β
NDRG1: N-Myc downstream regulated gene 1
LRP6: Low-density lipoprotein receptor-related protein 6
OPN: Osteopontin
OSX: Osterix
PPARγ: Peroxisome proliferator-activated receptor γ
Runx2: Runt-related transcription factor 2
SGK-1: Serum/glucocorticoid regulated kinase 1
siRNA: Small interfering RNA
TCF7L2: Transcription factor 7 like 2
Wnt: Wingless-type MMTV integration site family
