# Supplementary material for: N-myc downstream regulated gene 1 suppresses osteoblast differentiation through inactivating Wnt/β-catenin signaling
Source: Stem Cell Res Ther. 2022 Feb 4;13:53. doi: 10.1186/s13287-022-02714-5 (PMC8817551; doi:10.1186/s13287-022-02714-5)
Supplement: Supplementary file 1 — Additional file 1. Supplementary tables 1 and 2. [file 13287_2022_2714_MOESM1_ESM.pdf]

Table S1. Primers used for RT-PCR and cloning

Genes	Forward primer sequences:	Reverse primer sequences
Pparγ	CTTGACAGGAAAGACAACGG	GCTTCTACGGATCGAAACTG
C/ebpα	CTGATTCTTGCCAAACTGAG	GAGGAAGCTAAGACCCACTAC
Fabp4	AAATCACCGCAGACGACAGG	GGCTCATGCCCTTTCATAAAC
adipsin	TGATGTGTGCAGAGAGCAAC	CGTAACCACACCTTCGACTG
Runx2	TCCTGTAGATCCGAGCACCA	CTGCTGCTGTTGTTGCTGTT
Alp	CCAGAAAGACACCTTGACTGTGG	TCTTGTCCGTGTCGCTCACCAT
Osterix	GGCTTTTCTGCGGCAAGAGGTT	CGCTGATGTTTGCTCAAGTGGTC
Osteocalcin	GCAATAAGGTAGTGAACAGACTCC	CCATAGATGCGTTTGTAGGCGG
Bsp	AATGGAGACGGCGATAGTTCCG	GGAAAGTGTGGAGTTCTCTGCC
Opn	GCTTGGCTTATGGACTGAGGTC	CCTTAGACTCACCGCTCTTCATG
β-actin	AAGACCTCTATGCCAACACAG	GGAGGAGCAATGATCTTGATC
Ndrg1 primers for cloning	TTGGTACCGAGCTCGGATCCGCCACCA TGTCCCGAGAGCTACATGA	GCTGGATATCTGCAGAATTCTTAGC AGGACACCTCCATGG
Ndrg1 shRNA oligo pair	GATCCGCAGGAGATCACACAACATTT CAAGAGAATGTTGTGTGATCTCCTGC TTTTTTACGCGTG	AATTCACGCGTAAAAAAGCAGGAG ATCACACAACATTCTCTTGAAATGTT GTGTGATCTCCTGCG

Table S2. siRNA sequences used for gene silencing

Genes	Sense sequences	Antisense sequences
Ndrg1 siRNA	GCUGUGGUGGAAUGCAAUUTT	AAUUGCAUUCCACCACAGCTT
β-catenin siRNA	GGACCUACACUUAUGAGAATT	UUCUCAUAAGUGUAGGUCCTT
