# Supplementary material for: N-myc downstream regulated gene 1 suppresses osteoblast differentiation through inactivating Wnt/β-catenin signaling
Source: Stem Cell Res Ther. 2022 Feb 4;13:53. doi: 10.1186/s13287-022-02714-5 (PMC8817551; doi:10.1186/s13287-022-02714-5)
Supplement: Supplementary file 2 — Additional file 2. Supplementary figures 1 and 2. [file 13287_2022_2714_MOESM2_ESM.pdf]

Figure S1

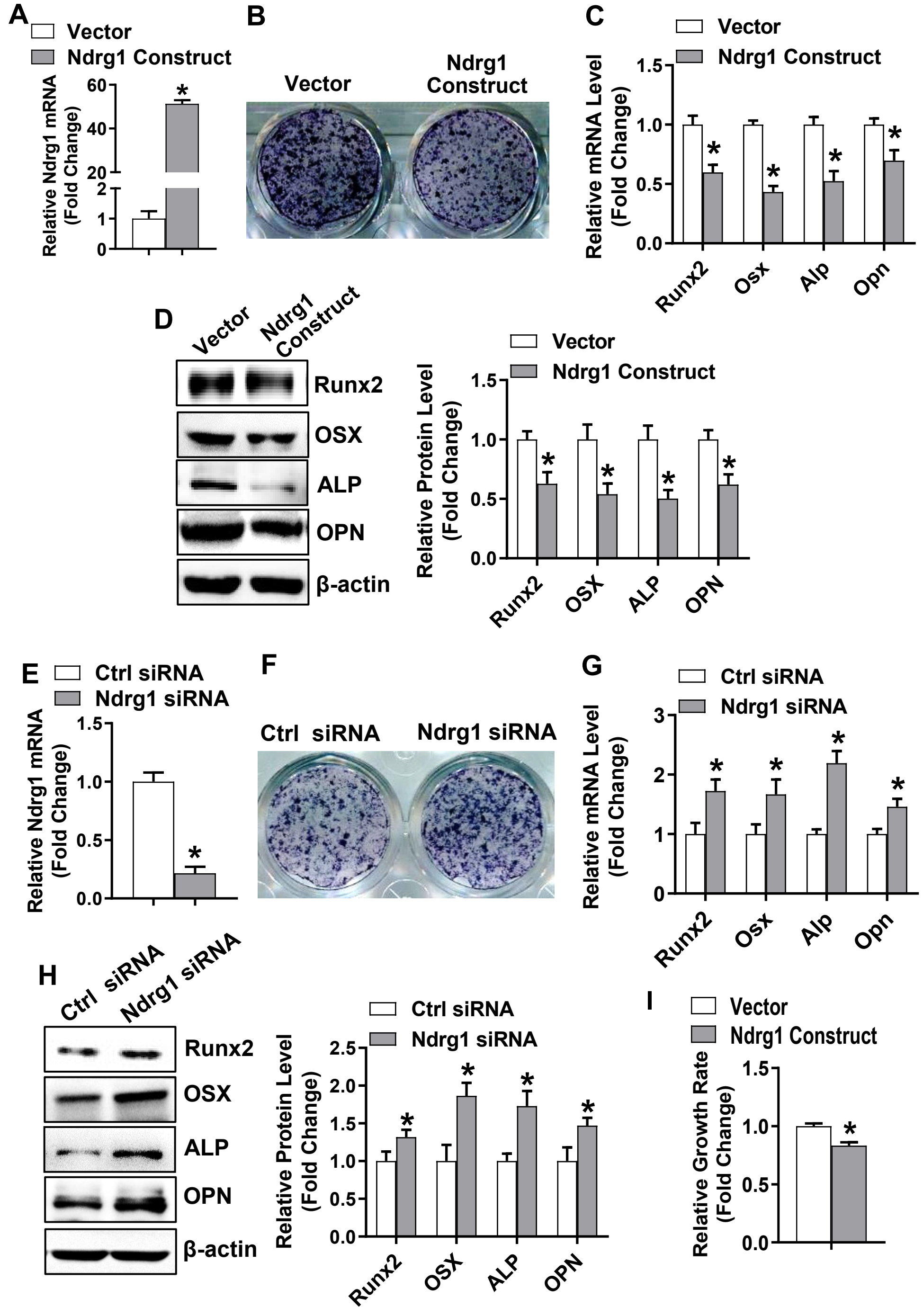

Figure S1. NDRG1 suppressed osteogenic differentiation of ST2 cells. Overexpression (A) or silencing (E) of Ndrg1 in ST2 cells after transfection of Ndrg1 expression construct or siRNA was verified using qRT-PCR. The cells following transfection were induced to allow osteogenic differentiation. The effects of Ndrg1 overexpression (B-D) or silencing (F-H) on osteogenic differentiation were examined. ALP staining was performed in differentiated osteoblasts 14 days after osteogenic treatment (B, F). The mRNA (C, G) and protein (D, H) levels of osteogenic factors were measured 72 h after osteogenic treatment. The effect of Ndrg1 overexpression on the cell growth rate was assayed (I). Values represent mean ± SD. n=3 in (A-H), n=5 in (I). *Significant *vs.* vector or control siRNA, $p < 0.05$.

Figure S2

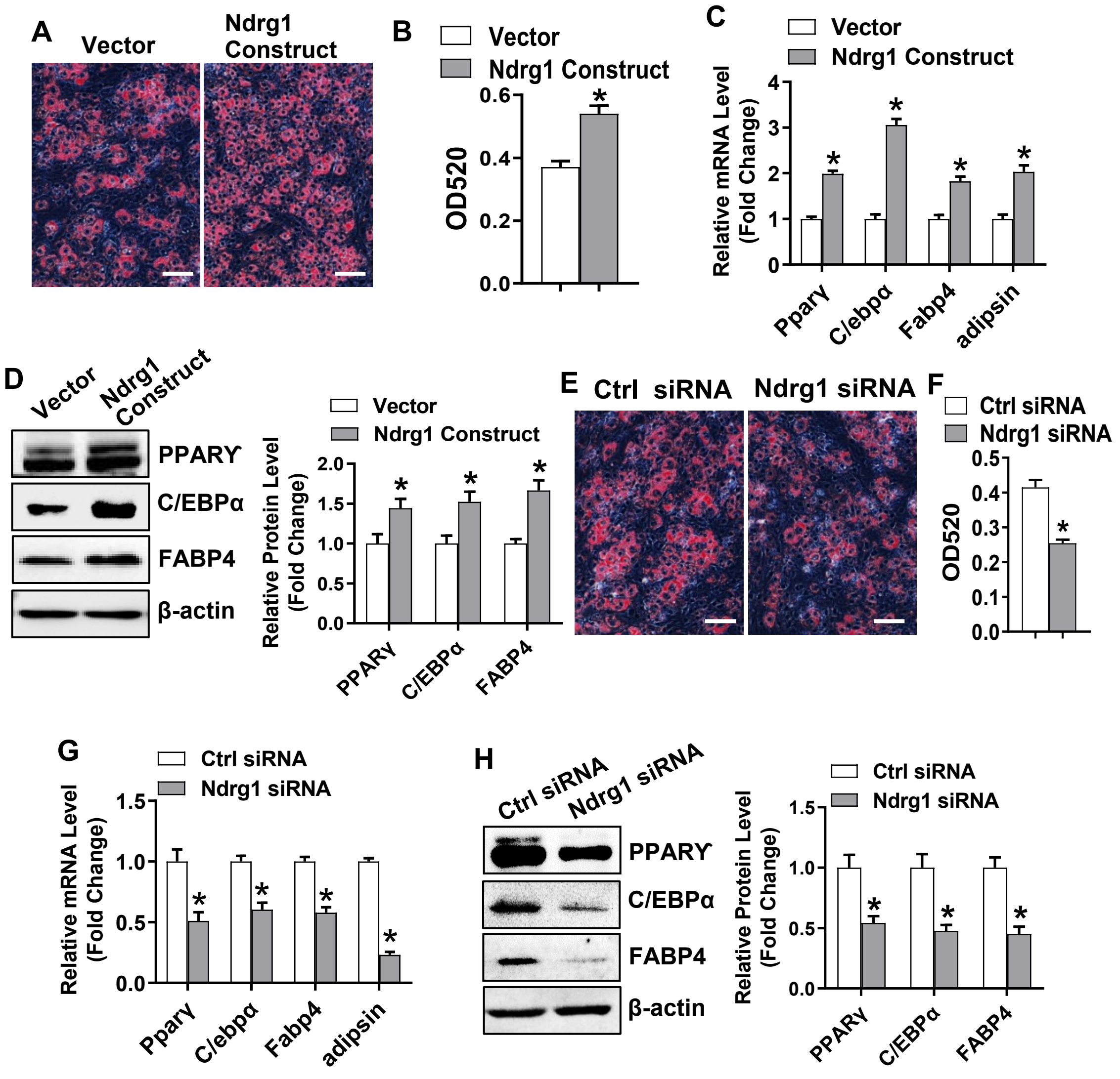

Figure S2. NDRG1 stimulated adipogenic differentiation of ST2 cells. The effects of Ndrg1 overexpression (A-D) or silencing (E-H) on adipogenic differentiation of ST2 cells were examined. The transfected cells were induced to allow adipogenic differentiation. Oil-red O staining was performed in differentiated adipocytes 5 days after adipogenic treatment (A, E) and the stain extracted with isopropanol was measured at 520 nm by spectrophotometry (B, F). The mRNA (C, G) and protein (D, H) levels of adipogenic factors were measured 48 h and 72 h, respectively, after adipogenic treatment. Image scale in (A) and (E): 100 μm. Values represent mean ± SD, n=3. *Significant vs. vector or control siRNA, $p < 0.05$.
